# Molecular insights unlocking therapeutic potential for multiple myeloma and bone disease management

**DOI:** 10.1186/s13046-024-03248-9

**Published:** 2024-12-18

**Authors:** Tiziana Bruno, Valeria Catena, Giovanni Blandino, Maurizio Fanciulli, Silvia Di Agostino

**Affiliations:** 1 SAFU Laboratory, Department of Research, Advanced Diagnostics, and Technological Innovation, Translational Research Area, IRCCS Regina Elena National Cancer Institute, Rome, Italy; 2 Translational Oncology Research Unit, IRCCS Regina Elena National Cancer Institute, Via Elio Chianesi, 53, 00144 Rome, Italy; 3 Department of Health Sciences, Magna Graecia University of Catanzaro, 88100 Catanzaro, Italy

**Keywords:** Multiple myeloma, Che-1, TAZ, microRNA, Microenvironment, Bone, Therapy

## Abstract

Multiple myeloma (MM), a hematologic malignancy characterized by the clonal expansion of plasma cells within the bone marrow, is associated with severe health complications, including osteolytic bone lesions that significantly increase the risk of fractures, leading to higher morbidity and mortality rates. One intriguing protein in this context is the RNA polymerase binding factor Che-1/AATF (Che-1), which has emerged as a potential player in the survival and proliferation of myeloma cells. Hippo pathway has been shown to be an important mediator of oncogenesis in solid tumors, especially for its role in shaping a tumor microenvironment favorable to cancer maintenance and spread. The Hippo pathway is also implicated in the pathogenesis of the osteolytic lesions that occurs in MM, since it deregulates the activities of mesenchymal populations of the bone matrix. In this commentary we wish to highlight some new molecular aspects elucidated in the paper by Bruno et al. regarding the proliferation of MM and the onset of bone lesions [Leukemia 38:877–882, 1]. A series of recent findings has revealed a crosstalk between the RNA polymerase binding factor Che-1 and the HIPPO downstream co-transcriptional factor TAZ, bringing to light new emerging molecular targets in MM to limit the development of bone lesions.

## Background

Multiple myeloma (MM) is a hematologic malignancy characterized by the proliferation of clonal plasma cells in the bone marrow [1]. This neoplasm is responsible for 10-15% of all hematologic cancers and accounts for approximately 20% of cancer-related deaths. Patients with MM often present with various clinical manifestations including anemia, hypercalcemia, renal impairment, compromised immune function, and particularly osteolytic bone lesions that leads to an increased risk of fractures, contributing to heightened morbidity and mortality [1]. The development of bone lesions in MM patients is the consequence of the alteration of the bone remodeling mechanism. In MM tumors, plasma cells release factors that stimulate osteoclast activity and inhibit osteoblast activity, leading to the degradation of bone tissue without adequate regeneration. The result is an imbalance between the activities of the two cell populations that determines bone erosion [2].

Che-1/AATF (Che-1) is an important protein that plays a multiple role in cell biology, particularly in gene transcription regulation, in the cellular response to DNA damage, and adaptation to various forms of cellular stress [3]. Recently, a variety of studies have linked Che-1 expression to poorer prognostic outcomes also describing its potential role in MM progression [4].

The Hippo signaling pathway is a particularly exciting area of research in both solid and hematologic tumors [5, 6]. The role of two co-transcriptional factors downstream of Hippo pathway, YAP/TAZ, is pivotal in regulating cellular differentiation and fate, especially in the context of MM. Although they act as oncogenes in many solid tumors, the role of YAP/TAZ in blood malignancies, including multiple myeloma, is still to be explored [7]. Interestingly, Grieve and colleagues reported that TAZ levels were lower in hematological malignancies, demonstrating that its promoter was hypermethylated in MM patient samples and cell lines [8]. The authors reported that the use of demethylating agents upregulated TAZ expression sensitizing MM cells to conventional antimyeloma therapies [9].

**Fig. 1:**
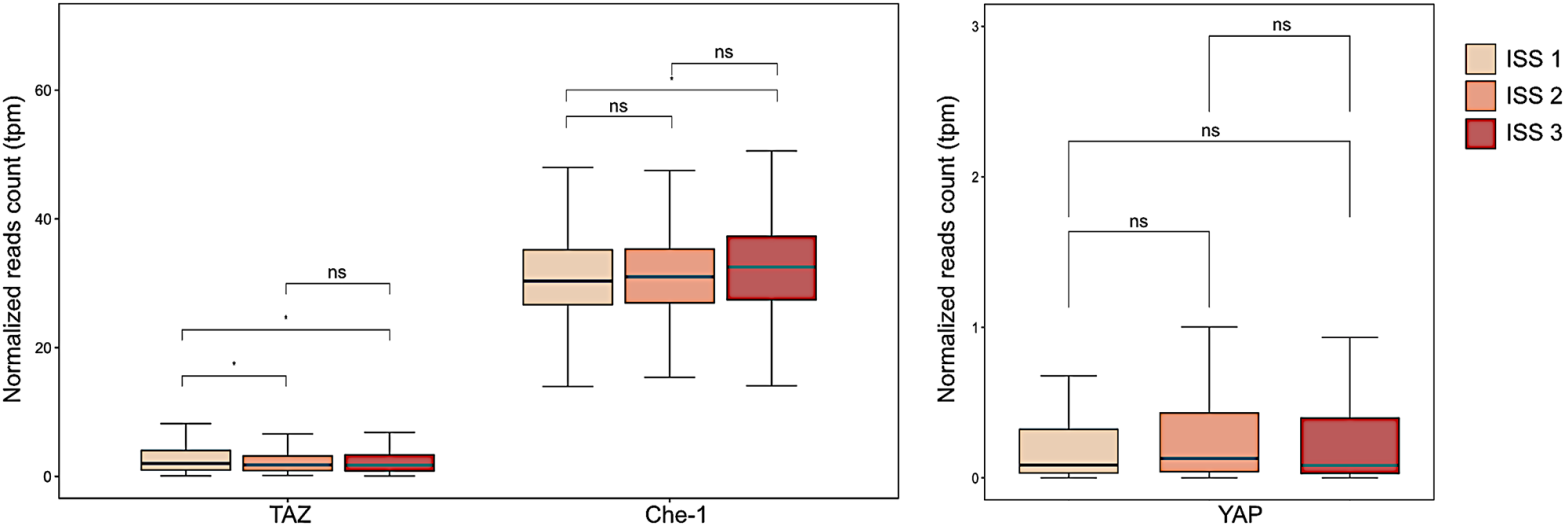
Box-plots showing the enrichment of TAZ, Che-1 **(A)**, and YAP **(B)** gene expression by the three different ISS related to the selected CoMMpass patient cohort (*N* = 687). The y-axis reports the normalized reads counts derived from the transcriptome of each patient and are expressed as transcripts per million (tpm) [1]

The recent study by Bruno and colleagues showed that TAZ is expressed at low levels in MM and is associated with poor patient outcomes, thus highlighting the anticorrelation between TAZ and Che-1 expression, both in the mouse transgenic model developing MM (Vk*Che-1) and in MM cell lines (Fig. 1A) [10]. Intriguingly, YAP expression does not influence MM development, aligning with studies that emphasize the independent roles of YAP and TAZ, as for example in lung cancer where TAZ was found to regulate principally genes involved in the remodeling of the extracellular matrix and therefore preside over processes of cell invasion and migration (Fig. 1B) [9]. Moreover, the ability of TAZ to promote osteoblastogenic differentiation and simultaneously inhibit adipogenic differentiation is particularly relevant in the bone marrow niche, where the balance between these cell types can impact disease progression and therapy resistance [10].

In this intricate antithetical crosstalk between Che-1 and TAZ, Bruno et al. described the role of miR-590-3p in MM progression and in the formation of osteolytic bone lesion. MicroRNAs (miRNAs) are shorts non-coding RNAs that play crucial roles in the regulation of gene expression by binding to complementary sequences in messenger RNAs (mRNAs). These interactions can lead to the degradation of target mRNAs or inhibit their translation, thus influencing various cellular processes [11]. In the context of MM, there is growing evidence that miRNA dysregulation is involved in the disease’s pathology [12].

In line with this, Bruno et al. proposed at least one mechanism parallel to that reported by Grieve et al., underscoring that Che-1 inhibits TAZ expression in MM at the post-transcriptional level, by inducing the transcription of miR-590-3p which in turn inhibits TAZ expression. Consistent with these results, elevated levels of miR-590-3p were found in various MM cell lines and transgenic mouse models (Vk*Che-1 and Vk*Myc) compared to control ones. This increase was also observed in patients with monoclonal gammopathy of undetermined significance (MGUS) and symptomatic disease. Also, the authors hypothesized that the reduced TAZ expression by miR-590-3p could also limit the osteoblastic differentiation by which an action on mesenchymal stem cells present in the bone marrow. To this aim, miR-590-3p was found to be released at elevated levels in the culture medium of MM cell lines panel compared to the levels from a lymphoblastoid cell line (LCL). Mesenchymal stem cells (MSCs) from Vk*Che-1 mice showed elevated miR-590-3p and reduced TAZ levels, indicating a potential interaction between these molecules in MM. Biochemical analyses showed increased bone resorption and decreased bone formation in Vk*Che-1 mice, underscoring the pathophysiological relevance of these findings. In vitro studies on human adipose-derived stem cells (ADSC1 and ADSC2) confirmed that osteogenic differentiation is linked to enhanced mineralization and upregulation of osteoblast markers, with increased TAZ expression and decreased Che-1 and miR-590-3p during differentiation.

**Fig. 2:**
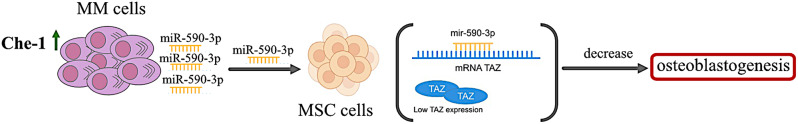
The illustration displays that high levels of Che-1 in MM cells promote miR-590-3p release in the microenvironment. This event in mesenchymal stem cells (MSC) can downregulate TAZ levels with concomitant inhibition of osteoblastogenesis process

However, overexpression of miR-590-3p in the medium negatively affected mineralization, leading to decreased TAZ and osteogenic marker expression. Che-1 overexpression in ADSC1 cells similarly results in increased miR-590-3p levels while downregulating TAZ and osteogenic differentiation markers (Fig. 2).

Overall, this study reveals the Che-1’s regulatory role in MM pathogenesis, thus suggesting that Che-1 modulates TAZ expression through miR-590-3p upregulation, also stressing the complex interplay between miRNAs and protein expression during osteoblast differentiation in MM and pointing out on potential therapeutic strategies.

Lastly, the preliminary data suggest that TAZ overexpression negatively regulates Che-1 expression, supporting the hypothesis of an inverse correlation between these proteins in MM.

## Conclusion

The recent paper by Bruno and colleagues along with other published studies partly discussed here, brought to light a growing body of knowledge on molecular mechanisms involving in transcriptional, post-transcriptional and epigenetic events in the maintenance of MM disease and associated with osteolytic lesions. Recently, much attention has been focused on potential diagnostic and prognostic markers for MM in liquid biopsy, including the detection of circulating cell-free DNAs, circulating miRNAs and circulating tumor cells [13]. Indeed, the levels of some circulating microRNAs from blood of MM patients have been associated with cancer proliferation and with the development of bone metastases [14]. Consistent with these findings, miR-590-3p could be a good candidate for detection in liquid biopsy. Multiple intracellular and extracellular events regulate the Hippo pathway and its regulatory mechanisms and aberrations in solid and blood tumors must still be thoroughly explored. Several research groups agree to claim that TAZ is downregulated in MM at multiple levels and these findings open a new window for cancer therapy in MM. Nanotechnology-based RNA delivery can greatly contribute to hamper the processes of cancer initiation and development and a good procedure could be represented by miR-590-3p suppression delivered by a nanocomplex inhibitor of mir-590-3p, also in co-treatment with demethylating agents to restore TAZ expression.

The ultimate goal should be the inhibition of AATF/Che-1 activity, which is highly expressed in several tumors. More recently, in metabolic dysfunction-associated steatohepatitis-driven hepatocellular carcinoma has been shown the efficacy of a tumor necrosis factor-alpha TNF-α inhibitor (Marimastat), a Che-1 antagonist [15, 16]. These and many other studies will help to better understand the mechanisms of carcinogenesis and metastasis to offer novel potential targets for therapeutic intervention.

## Data Availability

Materials can be obtained upon reasonable request from the corresponding author.
